# Occurrence of Granulomas in Bovines: An Abattoir-Based Study

**DOI:** 10.5402/2011/756087

**Published:** 2012-01-23

**Authors:** Sambath Uma, Madhavan Gopalakrishnan Nair, Khub Chandra Varshney

**Affiliations:** Department of Veterinary Pathology, Rajiv Gandhi College of Veterinary and Animal Sciences, Kurumbapet, Puducherry 605 009, India

## Abstract

The present study was carried out to record the occurrence of naturally occurring granulomas in cattle and buffaloes. Tissues grossly suspected for granulomas were collected from 336 out of 1600 (21%) abattoir cases. The gross features ranged from focal necrosis to large caseated masses, hard nodules, growths and abscesses. Histopathologically, 102 tissue samples (6.38%) were ascertained as granulomas. Majority of the granulomas were found in the liver 42 (41.18%), followed by lungs 22 (20.59%), lymph nodes 11 (10.78%), kidneys 6 (5.88%) and 14 (13.73%) as subcutaneous nodules/growths. Solitary cases were found in tongue, muscle, and urinary bladder, whereas 4 granulomas (3.92%) were found in body cavities. Based on the cellular component, the granulomas were categorized as epithelioid (53%), eosinophilic (37%) and suppurative (10%). Employing special staining techniques, the possible etiology of 75 granulomas could be identified. Among these, 70 granulomas (68.63%) were of infectious nature (parasitic 37 (36.28%), bacterial 32 (31.37%), and fungal 1(0.98%)). Non-infectious granulomas 5 cases (4.90%) included two lipid granulomas (1.96%), two granulomas (1.96%) associated with neoplasms and one (0.31%) associated with renal calculi. In 27 (26.47%) cases, the etiology of the granuloma could not be established and were categorized as granulomas of unknown etiology.

## 1. Introduction

Granulomas are tissue reactions typically elicited by persistent, indigestible pathogens, pathogen products, or inert foreign material. Granulomas are pathologically defined by the presence of macrophage-derived epithelioid cells and giant cells, whose organization can be modified by the cells of acquired immunity. The structural similarity in granulomas of diverse etiology suggests that a common set of pathophysiological signals regulates granulomatous inflammation. Observations that granulomas are of differing morphology and caused by a wide variety of irritants have led to numerous attempts to classify granulomatous inflammation in man, either to help in the diagnosis of granulomatous disease or to further the understanding of the granulomatous process [1]. However, in animals, there are limited studies on naturally occurring granulomas and very few attempts have been made to record and classify naturally occurring granulomas in large animals [2, 3]. The objective of the present study was to record the occurrence of granulomas in various tissues and organs in slaughtered cattle and buffaloes.

## 2. Materials and Methods

### 2.1. Period of Study

The collection of the samples for the study was carried out during the period from June 2006 to April 2007.

### 2.2. Collection and Processing of Samples

Organs/tissue samples with gross features of granuloma (ranging from necrotic spots, hard nodules, and growths to abscesses) were obtained from various abattoirs in India located at Puducherry, Chennai, Bangalore, Namakkal, and Kanpur.

A detailed gross examination of the lesion with respect to the size, shape, color, and consistency was carried out. Representative samples of the tissues were fixed in 10% neutral buffered formalin for routine paraffin embedding and microtomy. From the fixed and processed tissues, 4-5 *μ*m thick sections were stained with haematoxylin and eosin [4]. After histological assessment, adjacent sections of tissue samples with granulomas were stained using special staining techniques to ascertain the possible etiology. These included Giemsa's staining for general identification of bacteria, fungi, or parasites, Ziehl-Neelsen's/Auramine O staining for mycobacterium and Periodic Acid-Schiff's/Gridley's/Grocott's stain for fungal elements as per methods described by Luna [4].

### 2.3. Classification of Granulomas

All the granulomas were classified based on (a) main cellular component as: (i) epithelioid/macrophage rich granuloma, (ii) suppurative/pyogranuloma, or (iii) eosinophilic granuloma and (b) etiological basis as (i) infectious granulomas, (ii) noninfectious/foreign body granulomas, or (iii) granulomas of unknown etiology.

## 3. Results

In the present study, out of 1600 cattle and buffalo carcasses examined at different sources, 336 tissue samples (21%) were suspected for granulomas. The gross features were characterized by focal grayish-white necrotic spots, large caseated masses, hard nodules as well as growths and abscesses. Based on histopathological assessment, 102 tissue samples (6.38%) were ascertained to be granulomas (Table 1).

The maximum number of granulomas was recorded in the liver, and they accounted for 35.56% (42/102). Pulmonary granulomas were recorded in 29.41% cases (22 lungs and 8 associated lymph nodes). Granulomas were recorded in 6 kidneys (5.88%) and in three associated renal lymph nodes. In 14 cases, subcutaneous granulomas were observed as hard, white, encapsulated nodules in the intercostal spaces and in the brisket region. In four cases, growths found in the body cavities were found to be granulomas. These growths were (i) small nodules in the peritoneum, (ii) a case of multiple nodular lesions in the thoracic cavity attached to the pleura, (iii) a growth attached to the rib, and (iv) A mass attached to the peritoneal wall. Solitary cases of granuloma were recorded in the tongue, muscle, and urinary bladder.

 Histologically, based on the main cellular components involved, the granulomas in various tissues and organs were classified into epithelioid, eosinophilic, and suppurative granulomas. It was observed that majority of the granulomas were epithelioid in nature (53%). Eosinophilic granulomas accounted for 37% and suppurative granulomas for 10% of the granulomas.

Special staining techniques were employed to ascertain the possible etiology of the granulomas. Based on the histopathological findings and etiological identification, the granulomas were classified and are shown in Table 2.

Out of 102 granulomas recorded in various organs, the etiology of 75 granulomas could be identified. It was observed that 70 granulomas (68.63%) were of infectious nature; 5 cases (4.90%) were noninfectious and in 27 cases (26.47%) no definite cause could be attributed. The infectious causes identified were parasites (37 cases, 36.28%), bacterial (32 cases, 31.37%) and one case of a fungal granuloma (0.98%). Non-infectious causes identified were lipid granulomas (2 cases, 1.96%), neoplasm-associated granulomas (2 cases, 1.96%), and a granuloma associated with renal calculi (0.31%). 

The distribution and classification of granulomas recorded in various organs and tissues of slaughtered cattle and buffaloes are represented in Table 3. 

## 4. Discussion 

The results of the present study are in accordance with the findings of previous reports [2, 3]. Granulomas were histopathologically ascertained in 102 tissue samples (6.38%). Majority of the granulomas were found in the liver 42 (41.18%), followed by lungs 22 (20.59%), lymph nodes 11 (10.78%), kidneys 6 (5.88%), and 14 (13.73%) were in the form of subcutaneous nodules/growths. Solitary cases were found in the tongue, muscle, and urinary bladder, whereas 4 granulomas (3.92%) were found in body cavities. In a survey of naturally occurring bovine granulomas carried out at Tirupati (Andhra Pradesh, South India), Yadgirkar [2] recorded granulomas in 315/24,225 (1.3%) carcasses of cattle and buffaloes. Among the 315 granulomas recorded by Yadgirkar [2] 121 (38.41%) were found in the liver, 91 (28.88%) in lungs, 74 (23.49%) in lymph nodes, 28 (8.8%) in intestines, and one (0.31%) in the testis. 

The difference in the overall percentage occurrence of granulomas reported in the present study carried out at various locations in South India and North India and that reported by Yadgirkar [2] is attributed to the variation in the total number of carcasses examined in these two studies and also due to the different geographical locations in which these studies were carried out. Further, due to lack of reliable trace back animal identification system in the regions where the study was carried out, it was difficult to precisely trace the origin and the husbandry practices of the animals slaughtered in these locations. 

Out of 102 granulomas, the etiology of 75 granulomas could be identified. It was observed that 70 granulomas (68.63%) were of infectious nature; 5 cases (4.90%) were non-infectious, and in 27 cases (26.47%) no definite cause could be attributed. The infectious causes identified were parasites (37 cases, 36.28%), bacterial (32 cases, 31.37%) and one case of a fungal granuloma (0.98%). Non-infectious causes identified were lipid granulomas (2 cases, 1.96%), neoplasm-associated granulomas (2 cases, 1.96%), and a granuloma associated with renal calculi (0.31%). The 315 granulomas recorded by Yadgirkar [2] in cattle and buffaloes were categorized as parasitic (149, 47.30%), bacterial (62, 11.68%), fungal (1, 0.31%), and nonspecific (103, 32.69%). 

Etiopathological classification of the granulomas indicated that majority of the granulomas were epithelioid in nature (53%). Eosinophilic granulomas accounted for 37% and suppurative granulomas for 10% of the granulomas. 

Hepatic granulomas accounted for 41.18% of all the granulomas, and amongst these 42 granulomas, 26 (61.90%) were eosinophilic, 15 (35.71%) were epithelioid, and one (2.38%) was suppurative. Eosinophilic hepatic granulomas were either parasite induced or were reactions noticed along the migratory tracts of parasites. In a slaughter house-based study of hepatic pathology in bovines carried out at Puducherry, India, Nair et al. [3] recorded hepatic eosinophilic granulomas in 18% of the carcasses examined and suggested these granulomas to be of parasitic origin. In abattoir surveys carried out in Tanzania, Makundi et al. [5] reported the presence of *Schistosoma bovis* in 34% cattle livers. The prevalence and intensities of infection observed in the slaughtered cattle in that study were not related to age, sex and grade of the animals. Agrawal and Banerjee [6] reported that variations in the geographical areas have an important impact on the occurrence and pathogenesis of diseases of parasitic origin. 

Out of the 15 epithelioid hepatic granulomas recorded, 12 were due to hydatid cysts and the other three were of unknown etiology. Gram-negative bacteria were detected in a solitary case of suppurative granuloma in the liver. According to Skoric et al. [7], Gram-negative bacteria that cause granuloma in liver include *Brucella* sp., *Francisella* sp., *Pseudomonas* sp., *Salmonella* sp., *Yersinia* sp., and *Actinobacillus *sp. 

The liver is the prime repository for granulomatous inflammation. In humans, about 10% of the routine liver biopsy specimens demonstrate granulomas, and most systemic granulomatous diseases are found to involve the liver, at least to some extent [8, 9]. This may be due to the increased population of mononuclear phagocytes, including Kupffer cells and other macrophages in the liver. These cells serve as sentinels screening the blood for such stimuli as microorganisms, circulating antigens and immune complexes, inflammatory mediators, and partially degraded foreign substance and, then, through incompletely understood mechanisms assemble into granulomas [10]. 

Pulmonary granulomas were recorded in 22 cases (21.58%). Majority of these granulomas (15 cases, 14.7%) were Ziehl-Neelsen acid-fast-positive tuberculous epithelioid granulomas. Pleural involvement was seen in two cases. In addition to the lungs, most of the tuberculous lesions were confined to the bronchial and mediastinal lymph nodes (8 cases, 7.84%). Balani [11] reported an incidence of 10.97% (34/310) of granulomatous lesions in the lungs of slaughtered buffaloes, and 0.97% of these were tuberculous. Choudary [12] recorded tuberculous pneumonia in 2% (5/250) slaughtered cattle. Yadgirkar [2] reported an incidence of 28.8% (91/315) of granulomatous lesions in the lungs of slaughtered cattle and buffaloes, and 3.8% (12 cases) of these were tuberculous granulomas which also involved the lymph nodes. 

In the present study, Gram-positive bacteria and filamentous organisms could be demonstrated in solitary cases of suppurative and epithelioid lung granulomas, respectively. According to Skoric et al. [7], the common Gram-positive organisms associated with lung granulomas are *Corynebacterium *sp.,* Mycobacterium *sp.,* Nocardia *sp., and* Rhodococcus* sp. Histopathology of a solitary lipogranuloma observed in the lung was similar to endogenous lipid granulomatous pneumonia in a human case reported by Sato et al. [13]. However, in the present case, the source of the lipid could not be ascertained. 

In the present study, granulomas were observed in six kidneys (6/15, 35.29%) and 3 associated lymph nodes. Features of epithelioid granuloma were noticed in three kidneys. These included a case with tuberculosis, another case associated with renal calculi as well as in one case of unknown etiology. Suppurative renal granulomas were recorded in three cases. In one case, Gram-negative bacteria were identified and the other two were associated with lymphomas. According to Skoric et al. [7] Gram-negative organisms which have been implicated in granulomas are *Corynebacterium renale*,* Brucella *sp.,* Francisella *sp*., Pseudomonas *sp*., Salmonella *sp*., Yersinia *sp., and* Actinobacillus* sp. 

In the present study, 13.73% (14/102) granulomas were recorded in the subcutaneous tissues. In 11 subcutaneous eosiniophilic granulomas, *Onchocerca gibsoni* (a medium sized filarioid which usually inhabit subcutaneous tissues, ligaments and aponeuroses of large mammals mostly found in south-east Asia) could be demonstrated. The gross and histopathology of eosinophilic granulomas evoked by *O. gibsoni* recorded in this study are in accordance with those reported by other workers (Shastri [14] and Balachandran et al. [15]). Solitary cases of epithelioid lipogranuloma (characterized by multiple necrotic foci, multinucleated giant cells and cholesterol clefts), epithelioid acid-fast-positive tuberculous granuloma, and suppurative granuloma were also recorded in the subcutaneous tissue. 

Granulomas were also recorded from the body cavities. A peritoneal nodule showed an eosinophilic granulomatous reaction suspected to be parasite induced. The gross and histopathological features of a large growth attached to the peritoneal wall had features of actinomycosis. Cattle have a relatively high capacity to wall off septic foci within the peritoneal cavity. These infections are usually secondary to perforating injuries caused by bite wounds or foreign bodies [16]. In the present study, suppurative granuloma was seen in the thigh muscle of a cow. Suppurative myositis in muscle may sometimes be haematogenous in origin, but more often they result from inoculation or from an extension of a suppurative focus in adjacent structures such as joints, tendon sheaths, or lymph nodes [16]. 

The granulomatous inflammatory response is ubiquitous in pathology, being a manifestation of many infective, toxic, allergic, autoimmune, and neoplastic diseases and also conditions of unknown aetiology. Granulomas induced by members of Mycobacterium tuberculosis complex (MTC) group, *Schistosomes* sp., *Echinococcus *sp. and even *Capillaria* sp. may pose a potential zoonotic risk. Some of these lesions such as those caused by *Mycobacterium tuberculosis *complex (MTC) group., *Brucella* sp. may occur at abnormal locations and would probably be overlooked by the consulting veterinarian. In the case of tuberculosis, studies have indicated that confirmatory diagnosis of tuberculous granuloma were made only after histopathological evaluation, bacteriological culture, or polymerase chain reaction analysis of tissues collected from affected animals. Considering these aspects, all granulomas in ruminants should be thoroughly evaluated to determine the underlying etiological agent in order to formulate a control and eradication strategy. In this respect, granuloma submission programs have played a major role in eradication of *Mycobacterium bovis* in some developed countries [17]. 

Another important aspect of granuloma is the economic loss incurred by farmers in animals bearing granulomas. It is clear from the present study that hepatic granulomas account for the majority of the cases. It is likely that affected animals may not show any pathognomonic signs and the condition usually remains clinically undiagnosed. However, due to the hepatic involvement the productivity of the animal is likely to be reduced. Since, in all the cases of granulomas, the affected organs are to be condemned, this is also of much concern because of its potential to cause economic losses. Hence, continued research into granulomatous inflammation is essential, not only for its theoretical value, but also for its potential clinical, zoonotic, and economic implications. 

## Figures and Tables

**Table 1:** Granulomas recorded in various organs/tissues of cattle and buffaloes.

Organs/tissues	Suspected	Confirmed
Liver	100	42
Lung and Pleura	82	22
Kidney	17	6
Spleen	8	N.D.
Lymph node	65	11
Subcutaneous nodule/mass	40	14
Growths in body cavities	10	4
Muscle	3	1
Intestine	2	N.D.
Uterus	2	N.D.
Bile duct	1	N.D.
Skin	1	N.D.
Mammary gland	1	N.D.
Testis	1	N.D.
Tongue	1	1
Urinary bladder	1	1
Oesophagus	1	N.D.

Total	336	102

N.D.: not detected.

**Table 2:** Etio-pathological classification of granulomas recorded in various organs (*n* = 102).

Granuloma type	Cause						
	P	B	F	L	C	N	U
Eosinophilic	24	—	—	—	—	—	14
Epithelioid	12	27	1	2	1	—	11
Suppurative	1	5	—	—	—	2	2

Total	37	32	1	2	1	2	27

P: Parasitic, B: bacterial, F: fungal, L: lipid, C: calculi associated, N: neoplasm associated, U: unknown.

**Table 3:** Distribution and classification of granulomas in various organs and tissues of slaughtered cattle and buffaloes (*n* = 102).

Organs/tissues	Granuloma	Cause						
	Type	P	B	F	L	C	N	U
Liver (42)	Eosinophilic	13	—	—	—	—	—	13
	Epithelioid	12	—	—	—	—	—	3
	Suppurative	—	1	—	—	—	—	—

Lung (22)	Epithelioid	—	16	1	1	—	—	3
	Suppurative	—	1	—	—	—	—	—

Lymph node (11)	Epithelioid	—	8	—	—	—	—	2
	Suppurative	—	—	—	—	—	1	—

Kidney (6)	Epithelioid	—	1	—	—	1	—	1
	Suppurative	—	1	—	—	—	1	1

Subcutaneous nodules/mass (14)	Eosinophilic	11	—	—	—	—	—	
	Epithelioid	—	1	—	1	—	—	—
	Suppurative	1	—	—	—	—	—	—

Growths in body cavities (4)	Eosinophilic							1
	Epithelioid	—	1	—	—	—	—	1
	Suppurative	—	1	—	—	—	—	—

Soft tissue growths (3)	Epithelioid	—	—	—	—	—	—	1
	Suppurative	—	1	—	—	—	—	1

Total		37	32	1	2	1	2	27

P: parasitic, B: bacterial, F: fungal, L: lipid, C: calculi associated, N: neoplasm associated, U: unknown.
